# The neighborhood food environment modifies the association between infant feeding and childhood obesity

**DOI:** 10.1186/s12889-024-18755-9

**Published:** 2024-05-08

**Authors:** Christopher E. Anderson, Shannon E. Whaley, Michael I. Goran

**Affiliations:** 1 Division of Research and Evaluation, Public Health Foundation Enterprises (PHFE) WIC, a Program of Heluna Health, 13181 Crossroads Parkway N #540, City of Industry, CA 91746 USA; 2 Department of Pediatrics, Children’s Hospital Los Angeles, University of Southern California, Los Angeles, CA USA

**Keywords:** Infant feeding, Infant formula, Breastfeeding, Food environment, Child obesity

## Abstract

**Background:**

The Special Supplemental Nutrition Program for Women, Infants and Children (WIC) issues infant formula to infants who are not fully breastfed, and prior research found elevated obesity risk among children receiving lactose-reduced infant formula with corn syrup solids (CSSF) issued by WIC. This study was conducted to evaluate associations between a broader set of specialty infant formulas issued by WIC and child obesity risk, whether neighborhood context (e.g. neighborhood food environment) modifies associations, and whether racial/ethnic disparities in obesity are partly explained by infant formula exposure and neighborhood context.

**Methods:**

WIC administrative data, collected from 2013–2020 on issued amount (categorical: fully formula fed, mostly formula fed, mostly breastfed, fully breastfed) and type of infant formula (standard cow’s milk formula, and three specialty formulas: any CSSF, any soy-based formula, and any cow's milk-based formula with added rice starch) and obesity at ages 2–4 years (defined as a Body Mass Index z-score ≥ 95th percentile according to World Health Organization growth standard) were used to construct a cohort (*n* = 59,132). Associations of infant formula exposures and race/ethnicity with obesity risk were assessed in Poisson regression models, and modification of infant feeding associations with obesity by neighborhood context was assessed with interaction terms.

**Results:**

Any infant formula exposure was associated with significantly higher obesity risk relative to fully breastfeeding. Receipt of a CSSF was associated with 5% higher obesity risk relative to the standard and other specialty infant formulas (risk ratio 1.05, 95% confidence interval 1.02, 1.08) independent of breastfeeding duration and receipt of other specialty infant formulas. The association between CSSF and obesity risk was stronger in neighborhoods with healthier food environments (10% higher risk) compared to less healthy food environments (null). Racial/ethnic disparities in obesity risk were robust to adjustment for infant formula exposure and neighborhood environment.

**Conclusions:**

Among specialty infant formulas issued by WIC, only CSSFs were associated with elevated obesity risk, and this association was stronger in healthier food environments. Future research is needed to isolate the mechanism underlying this association.

## Background

Childhood obesity prevalence in the United States is substantial [1], and the prevalence of severe obesity continues to increase [2]. Many dietary behaviors in infancy are protective against obesity including receiving breastmilk instead of infant formula [3], breastfeeding instead of bottle feeding [4], receiving a lactose-based infant formula instead of a glucose-based infant formula [5], and recommended timing of introduction of complementary foods and beverages instead of earlier than recommended introduction [6, 7]. Children who are breastfed for longer exhibit fewer adverse complementary feeding behaviors that contribute to elevated obesity risk relative to formula fed infants, such as early introduction of solids [8]. Infant feeding practices, such as breastfeeding, are socially patterned [9], and lower rates of breastfeeding among low-income [10] and racial/ethnic minority [11] children may contribute to disparities in obesity risk among children [12]. Similarly, neighborhood contexts including the food environment, deprivation, and opportunity, contribute to obesity risk and socioeconomic and racial/ethnic disparities in obesity risk among children [13]. Obesity is highly prevalent among Special Supplemental Nutrition Program for Women, Infants, and Children (WIC)-participating children [14, 15], and WIC-participating children who are Hispanic have substantially higher obesity prevalence relative to other racial/ethnic groups in Southern California and nationally [15, 16].

The WIC program is a nutrition assistance program of the federal government of the United States that provides supplemental food packages, nutrition education, breastfeeding support, and health and social service referrals to pregnant, breastfeeding, or postpartum women and their infants and children to age 5 years among households with income < 185% of the Federal Poverty Level (FPL) [17, 18], assisting over 6.2 million participants per month in 2020 [19] and over 57% of eligible individuals [20]. Among WIC-participating children, longer duration of exclusive [21] and any breastfeeding [22] is associated with reduced risk of obesity. Despite WIC breastfeeding promotion, a majority of participating infants begin receiving infant formula by the second month of life [23].

A recent analysis among WIC participants in Southern California identified an association between receipt of a lactose-reduced infant formula made with corn syrup solids (CSSF) and increased obesity risk [5], which is hypothesized to occur via glycemic programming of the metabolism (in response to a glucose-based formula as opposed to lactose-based formula) [24], alteration of the infant gut microbiome [25], or the development of taste preferences. Whether the other specialty infant formulas issued by WIC programs contribute to elevated obesity risk, whether the neighborhood context of WIC participants contributes to these associations between infant formula and obesity risk, and whether different types of infant formula contribute to racial/ethnic disparities in obesity risk remains unknown. This study aims to assess whether infant feeding, including the amount of breastfeeding/formula feeding and each type of infant formula received by WIC-participating children, are associated with obesity risk, and whether these associations are modified by the neighborhood food environment. Further, we aim to assess whether infant feeding practices and neighborhood context contribute to racial/ethnic disparities in obesity among WIC-participating children. It was hypothesized that the association between infant feeding (amount and type of formula) with increased obesity risk would be stronger in less healthy (i.e. higher density of unhealthy food outlets, lower density of healthy food outlets) food environments, and that infant feeding and the neighborhood food environments would contribute to racial/ethnic disparities in obesity risk among WIC participants.

## Methods

### Subjects and setting

Administrative data from WIC participants served by one large local agency WIC program in Los Angeles County (LAC), California between January 2013 and March 2020 were used in this study. WIC administrative data contain information on the dates of service, child length (or height) and weight measurements, race/ethnicity, age, sex, and the food package issued by WIC each month; maternal language preference and educational attainment; and household size, number of WIC participating individuals, income, Supplemental Nutrition Assistance Program participation, and Medicaid participation. Only children with data on the WIC infant package issued every month for at least 12 months between 0 and 12 months of age, a geocoded residence in LAC to allow linkage to neighborhood contextual factors, a length and weight measurement before 6 months of age and a height and weight measurement after 24 months of age were included in this study (*n* = 59,132).

### Exposure

Infant feeding practices were the primary exposure of interest in this study, and WIC infant packages issued from 0 to 12 months of age were used to characterize these exposures. Infants are issued one of four infant packages by WIC each month to provide sufficient infant formula to complement maternally reported breastfeeding and meet the nutritional needs of participating infants: a fully breastfeeding infant package (0 mL of infant formula monthly), a mostly breastfeeding infant package (≤ 5,323 mL of infant formula monthly), a some breastfeeding infant package (6,624 to 11,918 mL of infant formula monthly), and a no breastfeeding infant package (9,927 to 13,071 mL of infant formula monthly) [26]. WIC infant package issuance data have previously been validated as a proxy for infant feeding practices [27], which is also supported by the large majority of families issued formula that redeem 100% of issued formula [28], and the 38% and 53% of participants in a national cohort of WIC-participating children reported that WIC provided too little formula to meet their child’s needs at < 6 months and 6–12 months of age, respectively [29]. Infant feeding was characterized by assessing the total amount of formula an infant was issued by WIC by adding 3 points for each month of fully breastfeeding package issuance, 2 points for each month of mostly breastfeeding package issuance, 1 point for each month of some breastfeeding package issuance, and 0 points for each month of no breastfeeding package issuance, multiplying scores for infants with only 12 months of issuance by 13/12 (e.g. for an infant with 12 months of issuance and a breastfeeding score of 36, the scaled score would be calculated as (13/12)*36 = 39 points), and categorizing infant package exposure into 4 categories: fully formula feeding (0 points), mostly formula feeding (1 to < 19 points), mostly breastfeeding (19 to < 39 points) and fully breastfeeding (39 points) [8, 22, 30]. Cow’s milk-based infant formula was considered the standard infant formula issued by WIC. Any exposure to each type of specialty infant formula issued by WIC, including CSSF, Soy-based infant formula (SOYF), and cow’s milk-based infant formula made with added rice starch (ARSF) was assessed dichotomously (0 months, ≥ 1 month of specified type).

### Outcome

Lengths or heights and weights were measured by WIC staff during the administration of services in WIC sites, and these measurements have been previously reported to have high validity [31]. These anthropometric measurements were used to calculate World Health Organization (WHO) body mass index z-scores (BMIz) [32], with obesity defined for this analysis for each measurement collected at ≥ 24 months of age as a BMIz value ≥ 95th percentile of the sex and age-specific growth standard.

### Covariates

Neighborhood context was assessed with both a metric of opportunity for healthy development, the Child Opportunity Index [33], and neighborhood food environment. The food environment was operationalized using the density of healthy (supermarkets, larger grocery stores, supercenters and produce stores) and unhealthy (fast food restaurants, small grocery stores, and convenience stores) outlets per square mile within the census tract of residence, aligning with the categories of healthy and less healthy food retailers used in the calculation of the modified retail food environment index [34, 35]. Based on prior evaluations of the association of the food environment with obesity among WIC participants in LAC, and following an initial exploration of polynomials for densities of healthy and unhealthy food outlets (linear, quadratic, and cubic for each), the food environment was operationalized as the density of healthy (linear and quadratic) and unhealthy (linear) food outlets within the census tract of residence [36, 37]. Other covariates for this analysis, available from WIC administrative data, included child age, sex (male, female), preterm delivery (yes: < 37 weeks gestation, no: ≥ 37 weeks gestation), race/ethnicity-language preference (reported by the child’s caregiver: Asian, Black, Spanish-speaking Hispanic, English-speaking Hispanic, White, and Other), maternal educational attainment (completed < high school, completed high school, completed > high school), and household income (≥ 100% FPL, < 100% FPL).

### Statistical analysis

Participating children were characterized within infant feeding groups (fully breastfed, and among the mostly breastfed, mostly formula fed, and fully formula fed by receipt of any specialty infant formula) with means and standard deviations or frequencies and percentages.

To assess the association between any receipt of each specialty infant formula and obesity risk independent of maternal, child, and household characteristics previously found to be associated with obesity risk, adjusted regression models were run. Risk ratios (RR) and 95% confidence intervals (CI) for the association of any receipt of each specialty formula with obesity risk were determined using generalized estimating equation (GEE) Poisson regression with robust standard error estimation accommodating repeated observations of children and clustering in census tracts [38], adjusted for infant feeding category (fully breastfed, mostly breastfed, mostly formula fed, fully formula fed), receipt of the focal specialty infant formula (no formula, any receipt of the specific specialty formula, receipt of only other infant formula), any receipt of the two other specialty infant formulas, child age at each BMI assessment (linear, quadratic, and cubic), race/ethnicity, sex, preterm delivery, age at baseline length and weight measurement and baseline BMI z-score; maternal education; and household poverty.

To assess whether the association between any receipt of each specialty infant formula and obesity risk was modified by the neighborhood food environment independent of maternal, child, and household characteristics previously found to be associated with obesity risk, adjusted regression models were run. Assessment of effect modification of the association of each specialty formula with obesity by the food environment was determined in GEE Poisson regression models with robust standard error estimation, accommodating repeated observations of each child and clustering within census tracts of residence [38]. RR (95% CI) were estimated at 25th, 50th and 75th percentiles of healthy and unhealthy outlet density distributions in regression models that included a primary exposure for any receipt of the specified specialty formula (e.g. three categories: any-CSSF, no CSSF, and fully BF), and were adjusted for the infant feeding category (fully breastfed, mostly breastfed, mostly formula fed, fully formula fed), any receipt of the 2 other specialty infant formulas, child age at the BMI assessment (linear, quadratic, and cubic), race/ethnicity, sex, preterm delivery, age at baseline length and weight measurement and baseline BMI z-score; maternal education; household poverty; neighborhood characteristics including the nationally-normalized overall Child Opportunity Index, healthy food outlet density (linear and quadratic), unhealthy food outlet density, two-way interactions between healthy (linear and quadratic) and unhealthy food outlet densities, two-way interactions between any specialty infant formula receipt and healthy and unhealthy food outlet densities, and three-way interactions between any specialty infant formula receipt with the two-way interactions between healthy (linear and quadratic) and unhealthy food outlet densities. Prevalence percent (95% CI) for obesity were assessed in identically parameterized GEE linear risk regression models with robust standard error estimation [39].

In light of previously reported differences in obesity risk between racial/ethnic groups of WIC-participating children [15, 16], this study evaluated the contribution of infant feeding and neighborhood context to racial/ethnic disparities in obesity risk in sequentially adjusted GEE Poisson regression models, with robust standard error estimation and accommodating repeated observations of children and clustering within census tract of residence [38]. Model 1 included independent terms for child age at the BMI assessment (linear, quadratic, and cubic), race/ethnicity, sex, preterm delivery, age at baseline length and weight measurement and baseline BMI z-score; maternal education; and household poverty. Model 2 included terms for Model 1 independent variables and for infant feeding exposures (amount of breastfeeding: fully breastfed, mostly breastfed, mostly formula fed, fully formula fed; any receipt of a CSSF, SOYF or ARSF). Model 3 included terms for Model 1 independent variables and neighborhood context (food environment and Child Opportunity Index). Model 4 included terms for Model 1 independent variables, infant feeding exposures and neighborhood context. Model 5 included terms for Model 4 independent variables and interactions between infant feeding exposures and neighborhood context.

## Results

**Fig. 1:**
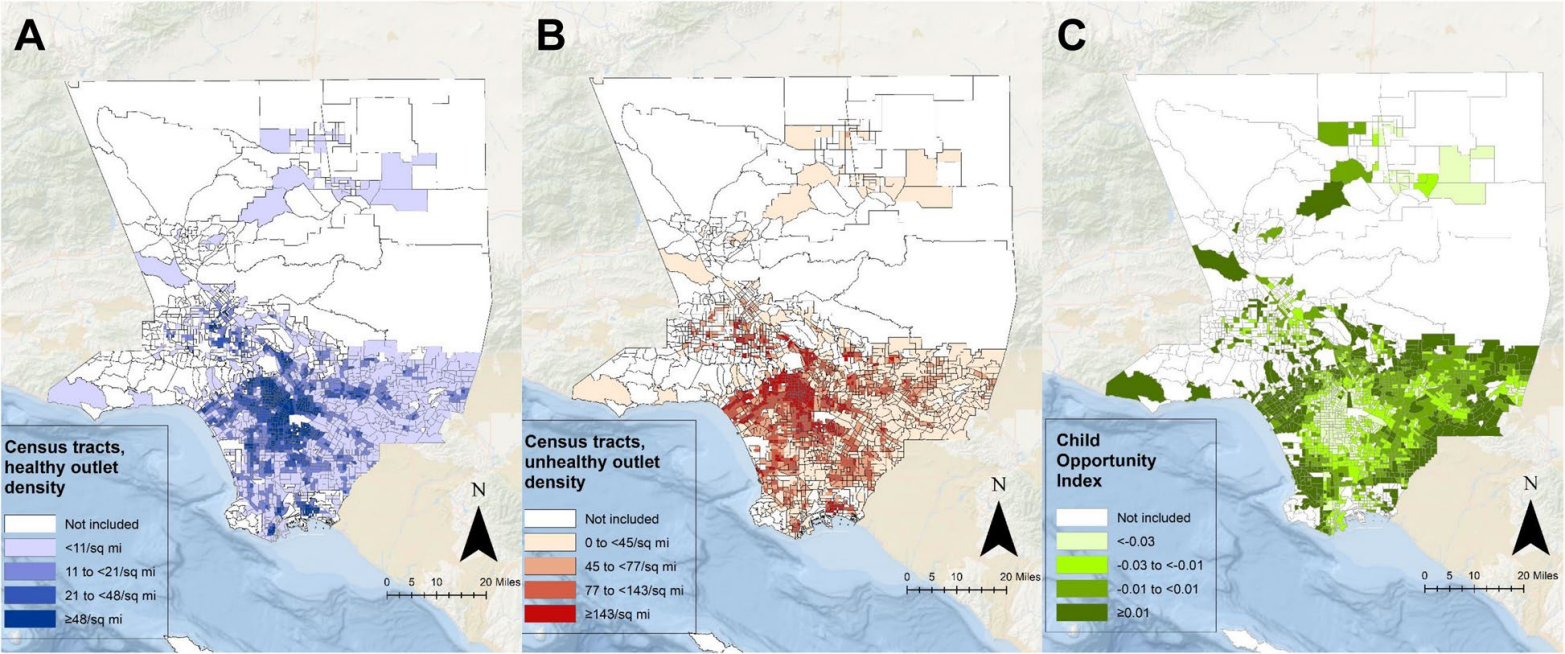
Quartiles of **A** healthy and **B** unhealthy food outlet densities and **C** Child Opportunity Index values among included census tracts in LA County, California

Of the 59,132 children, 79.1% received any infant formula between 0 and 12 months of age, with 33.2% receiving at least 1-month of a specialty infant formula (Table 1). Among recipients of any specialty infant formula, CSSFs were received for the greatest number of months, followed by standard formula, SOYF, and finally ARSF. Over half of participants were male, with male children overrepresented among recipients of any specialty formula. Black, White, and English-speaking Hispanic children were more likely to be recipients of any specialty infant formula than Asian and Spanish-speaking Hispanic children. Preterm delivery was more prevalent among all formula recipient categories than fully breastfed children, and was also more prevalent among specialty formula recipients than standard formula only recipients in each category for formula amount (mostly breastfed, mostly formula fed, fully formula fed). Parental educational attainment of greater than high school completion was more prevalent among specialty formula recipients, and specialty formula recipients had lower initial BMIz, higher last BMIz, and higher prevalence of obesity at the last measurement relative to infants who were fully breastfed or received no specialty formula. Healthy (Table 1, Fig. 1, panel A) and unhealthy (Table 1, Fig. 1, panel B) food outlet densities were lower in the neighborhoods of specialty formula recipients relative to non-recipients. No differences were observed for average Child Opportunity Index (Table 1, Fig. 1, panel C) between the neighborhoods of specialty formula recipients and non-recipients.

**Table 1:** Characteristics of WIC-participating children, by infant feeding and type of infant formula received, in LA County, California 2013–2020 (*n* = 59,132)

	**Fully breastfed**	**Mostly breastfed**		**Mostly formula fed**		**Fully formula fed**	
		**Standard formula only**	**Any specialty**	**Standard formula only**	**Any specialty**	**Standard formula only**	**Any specialty**
	*N* = 12,331	*N* = 8,002	*N* = 2,662	*N* = 15,485	*N* = 8,408	*N* = 7,764	*N* = 4,480
Infant formula issuance (mo), mean ± SD							
Standard cow’s milk formula	0.00 ± 0.00	9.14 ± 3.83	2.61 ± 3.30	11.74 ± 1.85	2.70 ± 3.41	12.33 ± 1.67	2.59 ± 3.38
CSSF	0.00 ± 0.00	0.00 ± 0.00	5.53 ± 4.32	0.00 ± 0.00	7.03 ± 4.75	0.00 ± 0.00	7.14 ± 5.07
ARSF	0.00 ± 0.00	0.00 ± 0.00	0.33 ± 1.59	0.00 ± 0.00	0.63 ± 2.30	0.00 ± 0.00	0.75 ± 2.55
SOYF	0.00 ± 0.00	0.00 ± 0.00	1.05 ± 2.73	0.00 ± 0.00	1.31 ± 3.23	0.00 ± 0.00	1.72 ± 3.78
Male	6189 (50.2)	4063 (50.8)	1387 (52.1)	8016 (51.8)	4453 (53.0)	3907 (50.3)	2317 (51.7)
Preterm delivery	599 (4.9)	426 (5.3)	191 (7.2)	1115 (7.2)	755 (9.0)	417 (5.4)	319 (7.1)
Race/ethnicity							
Asian	604 (4.9)	815 (10.2)	144 (5.4)	1477 (9.5)	361 (4.3)	752 (9.7)	163 (3.6)
Black	594 (4.8)	226 (2.8)	144 (5.4)	468 (3.0)	543 (6.5)	389 (5.0)	451 (10.1)
Hispanic-EN	5746 (46.7)	2535 (31.7)	1257 (47.4)	7241 (46.8)	5137 (61.2)	4289 (55.3)	2979 (66.7)
Hispanic-SP	4595 (37.3)	4194 (52.5)	966 (36.4)	5870 (38.0)	1987 (23.7)	2133 (27.5)	701 (15.7)
White	511 (4.2)	128 (1.6)	88 (3.3)	185 (1.2)	218 (2.6)	73 (0.9)	94 (2.1)
Other	262 (2.1)	91 (1.1)	54 (2.0)	225 (1.5)	154 (1.8)	116 (1.5)	81 (1.8)
Household income < 100% FPL	8878 (72.0)	6103 (76.3)	1940 (72.9)	12061 (77.9)	6387 (76.0)	6267 (80.7)	3604 (80.4)
Parental educational attainment							
< HS completion	2330 (18.9)	2254 (28.2)	467 (17.5)	3441 (22.2)	1299 (15.4)	1526 (19.7)	700 (15.6)
Completed HS	2347 (19.0)	1664 (20.8)	511 (19.2)	3703 (23.9)	1730 (20.6)	2130 (27.4)	1115 (24.9)
> HS completion	7654 (62.1)	4084 (51.0)	1684 (63.3)	8341 (53.9)	5379 (64.0)	4108 (52.9)	2665 (59.5)
Child anthropometry, mean ± SD							
Initial BMIz	0.53 ± 1.23	0.48 ± 1.29	0.31 ± 1.29	0.44 ± 1.26	0.30 ± 1.24	0.51 ± 1.20	0.37 ± 1.29
Age (y) at initial BMIz	0.35 ± 0.21	0.28 ± 0.20	0.26 ± 0.19	0.28 ± 0.20	0.27 ± 0.20	0.28 ± 0.20	0.27 ± 0.20
Last BMIz	0.76 ± 1.18	0.91 ± 1.28	0.93 ± 1.29	1.14 ± 1.41	1.19 ± 1.43	1.16 ± 1.46	1.22 ± 1.45
Age (y) at last BMIz	3.49 ± 0.89	3.56 ± 0.89	3.39 ± 0.90	3.51 ± 0.90	3.35 ± 0.90	3.39 ± 0.89	3.24 ± 0.89
Obese at last measurement, n (%)	2203 (17.9)	1741 (21.8)	622 (23.4)	4418 (28.5)	2520 (30.0)	2250 (29.0)	1368 (30.5)
Neighborhood variables, mean ± SD							
Healthy food outlets/10 per miles^2^	4.67 ± 6.56	5.81 ± 7.65	4.29 ± 6.11	5.22 ± 7.18	3.59 ± 5.07	3.79 ± 5.22	2.93 ± 4.12
Unhealthy food outlets/10 per miles^2^	15.73 ± 23.78	18.52 ± 26.89	14.02 ± 20.63	15.92 ± 23.42	11.43 ± 16.36	10.98 ± 1.57	9.13 ± 12.63
Child Opportunity Index	-0.02 ± 0.02	-0.02 ± 0.02	-0.02 ± 0.02	-0.02 ± 0.02	-0.02 ± 0.02	-0.02 ± 0.02	-0.02 ± 0.02

*ARSF* Cow’s milk based formula with added rice starch, *BMIz* Body mass index z-score, *CSSF* Lactose-reduced infant formula with corn syrup solids, *EN* English-speaking, *FPL* Federal poverty level, *HS* High school, *LA* Los Angeles, *mo* Months, *SD* Standard deviation, *SOYF* Soy-based formula, *SP* Spanish-speaking, *WIC* The Special Supplemental Nutrition Program for Women, Infants, and Children, *y* Years

Associations between any receipt of each of the three specialty infant formulas and obesity risk were assessed (Table 2). Obesity risk was 64% higher among CSSF recipients (RR 1.64, 95% CI 1.57, 1.71), 58% higher among SOYF recipients (RR 1.58, 95% CI 1.49, 1.68) and 51% higher among ARSF recipients (RR 1.51, 95% CI 1.39, 1.63) relative to fully breastfed infants. When recipients of each specialty infant formula were compared to infants receiving only the other three infant formulas (standard and the two other specialty), CSSF recipients exhibited 5% higher obesity risk (RR 1.05, 95% CI 1.02, 1.08) than recipients of non-CSSF infant formulas (standard, SOYF and ARSF).

**Table 2:** Association of receipt of each specialty contract infant formula with obesity risk at 2–4 years of age among WIC-participating infants in LA County, California 2013–2020 (*n* = 59,132)

Specialty formula type^a^	Comparison to fully breastfed^b^			Specific specialty vs Standard/other specialty only^b^
	Fully breastfed	Specific specialty^a^	Standard/other specialty only^a^	
CSSF	1.00 (ref)	**1.64 (1.57, 1.71)**	**1.58 (1.52, 1.63)**	**1.05 (1.02, 1.08)**
SOYF	1.00 (ref)	**1.58 (1.49, 1.68)**	**1.58 (1.52, 1.63)**	1.02 (0.96, 1.07)
ARSF	1.00 (ref)	**1.51 (1.39, 1.63)**	**1.58 (1.52, 1.63)**	0.97 (0.90, 1.04)

*ARSF* Cow’s milk based formula with added rice starch, *CSSF* Lactose-reduced infant formula with corn syrup solids, *LA* Los Angeles, *SOYF* Soy-based formula, *WIC* The Special Supplemental Nutrition Program for Women, Infants, and Children

^a^Specialty infant formula receipt was defined as a 3-category variable for each formula type: any of the specified specialty formula, only the other infant formulas, no infant formula. The fully breastfed group had *n* = 12,311 individuals in each model. For the CSSF model, the specialty formula group had *n* = 12,671 and other formula only had *n* = 34,130. For the SOYF model the any specialty formula group had *n* = 3,255 and the other formula only group had *n* = 43,546. For the ARSF model the any specialty formula group had *n* = 1,309 and the other formula only group had *n* = 45,492

^b^Risk ratios (95% confidence interval) for the association of receipt of each specialty infant formula with obesity risk were determined in generalized estimating equation Poisson regression models with robust standard error estimation. Models were adjusted for the infant feeding category (fully breastfed, mostly breastfed, mostly formula fed, fully formula fed), any receipt of the 2 other specialty infant formulas, child age at the BMI assessment (linear, quadratic, and cubic), race/ethnicity, sex, age at baseline length and weight measurement, preterm delivery, and baseline BMI z-score; maternal education; and household poverty

CSSF-receipt and receipt of only non-CSSF infant formulas were associated with elevated obesity risk relative to fully breastfeeding across the observed range of healthy and unhealthy food outlet densities, but the magnitude of association between CSSF-receipt and obesity risk decreased as unhealthy food outlet density increased (Table 3). Healthy and unhealthy food outlet densities significantly modified the relationship between CSSF-receipt and obesity risk (*p*-values 0.02 and 0.0001, respectively). CSSF-receipt was associated with elevated obesity risk relative to receipt of only other infant formulas (standard and other specialty formulas) at low unhealthy food outlet density regardless of healthy outlet density. At intermediate unhealthy outlet densities, CSSF-receipt was associated with elevated obesity risk only if healthy outlet density was intermediate to high. At high unhealthy outlet density, CSSF-receipt was not associated with obesity risk regardless of healthy outlet density.

**Table 3:** Association of any CSSF, no-CSSF and fully breastfed with obesity risk by food environment in the neighborhood of residence among WIC-participating children in LA County, California, 2013–2020 (*n* = 59,132)

Unhealthy outlet density^a^	Infant feeding group	Healthy outlet density^a^					
		11.0/mi^2^		21.0/mi^2^		48.0/mi^2^	
		% (95% CI)^b^	RR (95% CI)^c^	% (95% CI)^b^	RR (95% CI)^c^	% (95% CI)^b^	RR (95% CI)^c^
		Any-CSSF vs Fully BF^a^					
45.0/mi^2^	Any CSSF	27.1 (25.5, 28.7)	**1.64 (1.55, 1.73)**	27.6 (26.0, 29.3)	**1.65 (1.57, 1.73)**	29.0 (27.0, 31.1)	**1.68 (1.50, 1.88)**
	Fully BF	15.2 (13.6, 16.9)	1.00 (ref)	15.3 (13.6, 16.9)	1.00 (ref)	15.3 (13.4, 17.2)	1.00 (ref)
77.0/mi^2^	Any CSSF	26.7 (25.0, 28.3)	**1.60 (1.51, 1.69)**	27.2 (25.7, 28.8)	**1.60 (1.53, 1.69)**	28.7 (26.9, 30.6)	**1.63 (1.46, 1.81)**
	Fully BF	15.5 (13.8, 17.1)	1.00 (ref)	15.5 (13.9, 17.1)	1.00 (ref)	15.5 (13.7, 17.3)	1.00 (ref)
143.0/mi^2^	Any CSSF	25.8 (23.8, 27.8)	**1.51 (1.38, 1.65)**	26.4 (24.7, 28.2)	**1.51 (1.39, 1.65)**	28.1 (26.3, 29.9)	**1.52 (1.34, 1.73)**
	Fully BF	15.9 (14.1, 17.7)	1.00 (ref)	16.0 (14.3, 17.6)	1.00 (ref)	15.9 (14.3, 17.6)	1.00 (ref)
		No-CSSF vs Fully BF^a^					
45.0/mi^2^	No CSSF	25.4 (23.9, 26.9)	**1.58 (1.51, 1.65)**	25.6 (24.1, 27.1)	**1.58 (1.51, 1.65)**	26.1 (24.5, 27.7)	**1.57 (1.45, 1.70)**
	Fully BF	15.2 (13.6, 16.9)	1.00 (ref)	15.3 (13.6, 16.9)	1.00 (ref)	15.3 (13.4, 17.2)	1.00 (ref)
77.0/mi^2^	No CSSF	25.7 (24.2, 27.1)	**1.58 (1.51, 1.65)**	25.8 (24.4, 27.3)	**1.58 (1.51, 1.64)**	26.3 (24.8, 27.9)	**1.57 (1.45, 1.70)**
	Fully BF	15.5 (13.8, 17.1)	1.00 (ref)	15.5 (13.9, 17.1)	1.00 (ref)	15.5 (13.7, 17.3)	1.00 (ref)
143.0/mi^2^	No CSSF	26.3 (24.7, 27.9)	**1.57 (1.47, 1.69)**	26.4 (24.9, 27.9)	**1.57 (1.47, 1.68)**	26.8 (25.2, 28.3)	**1.57 (1.42, 1.73)**
	Fully BF	15.9 (14.1, 17.7)	1.00 (ref)	16.0 (14.3, 17.6)	1.00 (ref)	15.9 (14.3, 17.6)	1.00 (ref)
		Any-CSSF vs No-CSSF^a^					
45.0/mi^2^	Any CSSF	27.1 (25.5, 28.7)	**1.06 (1.01, 1.10)**	27.6 (26.0, 29.3)	**1.07 (1.03, 1.11)**	29.0 (27.0, 31.1)	**1.10 (1.04, 1.16)**
	No CSSF	25.4 (23.9, 26.9)	1.00 (ref)	25.6 (24.1, 27.1)	1.00 (ref)	26.1 (24.5, 27.7)	1.00 (ref)
77.0/mi^2^	Any CSSF	26.7 (25.0, 28.3)	1.03 (0.99, 1.08)	27.2 (25.7, 28.8)	**1.05 (1.01, 1.08)**	28.7 (26.9, 30.6)	**1.08 (1.03, 1.13)**
	No CSSF	25.7 (24.2, 27.1)	1.00 (ref)	25.8 (24.4, 27.3)	1.00 (ref)	26.3 (24.8, 27.9)	1.00 (ref)
143.0/mi^2^	Any CSSF	25.8 (23.8, 27.8)	0.99 (0.93, 1.05)	26.4 (24.7, 28.2)	1.00 (0.96, 1.06)	28.1 (26.3, 29.9)	1.04 (1.00, 1.09)
	No CSSF	26.3 (24.7, 27.9)	1.00 (ref)	26.4 (24.9, 27.9)	1.00 (ref)	26.8 (25.2, 28.3)	1.00 (ref)

*BF* Breastfed, *CI* Confidence interval, *CSSF* Lactose-reduced infant formula with corn syrup solids, *LA* Los Angeles, *RR* Risk ratio, *WIC* The Special Supplemental Nutrition Program for Women, Infants, and Children

^a^Prevalence percent (95% CI) and RR (95% CI) are presented at combinations of healthy and unhealthy food outlet density in the census tract of residence. Healthy and unhealthy outlet densities were chosen at the 25th, 50th, and 75th percentiles of the distribution of the respective distributions for outlet density

^b^Prevalence percent (95% CI) of obesity is presented for each infant feeding group at each specified combination of healthy and unhealthy food outlet density, estimated using generalized estimating equation linear risk regression models with robust standard error estimation and accommodating repeated observations of each child and clustering within census tract of residence. Models included a primary exposure for any receipt of the specified specialty formula (three categories: any-CSSF, no CSSF, and fully BF), and were adjusted for the infant feeding category (fully breastfed, mostly breastfed, mostly formula fed, fully formula fed), any receipt of the 2 other specialty infant formulas, child age at the BMI assessment (linear, quadratic, and cubic), race/ethnicity, sex, age at baseline length and weight measurement, preterm delivery, and baseline BMI z-score; maternal education; household poverty; neighborhood characteristics including the nationally-normalized overall Child Opportunity Index, healthy food outlet density (linear and quadratic), unhealthy food outlet density, two-way interactions between healthy (linear and quadratic) and unhealthy food outlet densities, two-way interactions between any CSSF receipt and healthy and unhealthy food outlet densities, and three-way interactions between any CSSF receipt with the two-way interactions between healthy (linear and quadratic) and unhealthy food outlet densities

^c^Risk ratios (95% confidence interval) for the association of receipt of each specialty infant formula with obesity risk were determined in generalized estimating equation Poisson regression models with robust standard error estimation, accommodating repeated observations of each child and clustering within census tracts of residence. Models included a primary exposure for any receipt of the specified specialty formula (three categories: any-CSSF, no CSSF, and fully BF), and were adjusted for the infant feeding category (fully breastfed, mostly breastfed, mostly formula fed, fully formula fed), any receipt of the 2 other specialty infant formulas, child age at the BMI assessment (linear, quadratic, and cubic), race/ethnicity, sex, age at baseline length and weight measurement and baseline BMI z-score; maternal education; household poverty; neighborhood characteristics including the nationally-normalized overall Child Opportunity Index, healthy food outlet density (linear and quadratic), unhealthy food outlet density, two-way interactions between healthy (linear and quadratic) and unhealthy food outlet densities, two-way interactions between any CSSF receipt and healthy and unhealthy food outlet densities, and three-way interactions between any CSSF receipt with the two-way interactions between healthy (linear and quadratic) and unhealthy food outlet densities. *P*-values indicate that associations between CSSF receipt and obesity risk was significantly modified by both healthy (combined interaction *p*-value = 0.02) and unhealthy (combined interaction *p*-value = 0.0001) food outlet densities

Racial/ethnic disparities in obesity risk were apparent before adjustment for infant feeding practices or neighborhood context (Table 4, minimally adjusted model), with 58% lower (RR 0.42, 95% CI 0.39, 0.45), 28% lower (RR 0.72, 95% CI 0.67, 0.78), 6% lower (RR 0.94, 95% CI 0.92, 0.97), 40% lower (RR 0.60, 95% CI 0.49, 0.74), and 24% lower (RR 0.76, 95% CI 0.68, 0.85) obesity risk for children in Asian, Black, Spanish-speaking Hispanic, White and Other race/ethnicity groups relative to English-speaking Hispanic children at 2–4 years of age. The disparity between Spanish-speaking Hispanic and English-speaking Hispanic groups dissipated following adjustment for infant feeding (infant feeding category and receipt of each type of infant formula), but no other disparities were meaningfully altered and all others persisted as significant following adjustment for infant feeding and neighborhood contextual factors.

**Table 4:** Disparities in risk of childhood obesity by race/ethnicity among WIC-participating children in LA County, California 2013–2020 (*n* = 59,132)

Race/ethnicity	Model 1:	Model 2:	Model 3:	Model 4:	Model 5:
	Minimally Adjusted^a^	Minimal Adj + INF^a^	Minimal Adj + NCon^a^	Minimal Adj + INF & NCon^a^	Minimal Adj + INF, NCon, interactions of INF & NCon^a^
Asian	**0.42 (0.39, 0.45)**	**0.43 (0.40, 0.46)**	**0.43 (0.40, 0.46)**	**0.43 (0.40, 0.46)**	**0.43 (0.40, 0.46)**
Black	**0.72 (0.67, 0.78)**	**0.73 (0.67, 0.78)**	**0.71 (0.66, 0.77)**	**0.72 (0.67, 0.77)**	**0.72 (0.67, 0.77)**
Hispanic, SP	**0.94 (0.92, 0.97)**	1.01 (0.98, 1.04)	**0.92 (0.90, 0.95)**	0.99 (0.96, 1.01)	0.99 (0.96, 1.01)
White	**0.60 (0.49, 0.74)**	**0.65 (0.52, 0.82)**	**0.63 (0.51, 0.77)**	**0.66 (0.53, 0.83)**	**0.66 (0.53, 0.83)**
Other	**0.76 (0.68, 0.85)**	**0.79 (0.71, 0.88)**	**0.77 (0.69, 0.86)**	**0.79 (0.71, 0.88)**	**0.79 (0.71, 0.88)**
Hispanic, EN	1.00 (ref)	1.00 (ref)	1.00 (ref)	1.00 (ref)	1.00 (ref)

*Adj* Adjusted, *CI* Confidence interval, *EN* English-speaking, *INF* Infant feeding, *LA* Los Angeles, *NCon* Neighborhood context, *RR* Risk ratio, *SP* Spanish-speaking, *WIC* The Special Supplemental Nutrition Program for Women, Infants, and Children

^a^All associations are RR (95% CI) estimated using generalized estimating equation Poisson regression models, with robust standard error estimation and accommodating repeated observations of children and clustering within census tract of residence. Model 1 was adjusted for child age at the BMI assessment (linear, quadratic, and cubic), race/ethnicity, sex, age at baseline length and weight measurement, preterm delivery, and baseline BMI z-score; maternal education; and household poverty. Model 2 was adjusted for Model 1 variables and any receipt of the specified specialty formula (three categories: any-CSSF, no CSSF, and fully BF), the infant feeding category (fully breastfed, mostly breastfed, mostly formula fed, fully formula fed), and any receipt of the 2 other specialty infant formulas. Model 3 was adjusted for Model 1 variables and neighborhood characteristics including the nationally-normalized overall Child Opportunity Index, healthy food outlet density (linear and quadratic), unhealthy food outlet density, and two-way interactions were included between healthy (linear and quadratic) and unhealthy food outlet density. Model 4 was adjusted for Model 3 variables and any receipt of the specified specialty formula (three categories: any-CSSF, no CSSF, and fully BF), the infant feeding category (fully breastfed, mostly breastfed, mostly formula fed, fully formula fed), and any receipt of the 2 other specialty infant formulas. Model 5 was adjusted for Model 4 variables and two-way interactions between any CSSF receipt and healthy and unhealthy food outlet densities, and three-way interactions between any CSSF receipt with the two-way interactions between healthy (linear and quadratic) and unhealthy food outlet densities

## Discussion

This study aimed to assess whether receipt of both standard and specialty infant formulas issued by WIC was associated with elevated risk of obesity, whether the neighborhood food environment modified these associations, and whether infant formula exposures and neighborhood food environments contributed to racial/ethnic disparities in obesity risk. Formula fed infants had higher risk of obesity than fully breastfed infants, regardless of infant formula type received. Formula fed infants who were issued at least one month of a CSSF had 5% higher obesity risk relative to formula fed infants issued only non-CSSF formulas. The neighborhood food environment modified the association between infant formula type and obesity risk, with stronger associations between issuance of a CSSF and elevated obesity risk observed in neighborhoods with healthier food environments (i.e. higher density of healthy food outlets and lower density of unhealthy outlets). Racial/ethnic disparities in obesity risk (significantly lower risk in all race/ethnicity groups compared to English-speaking Hispanic children) were apparent among study participants and were robust to adjustment for infant feeding and neighborhood context.

The associations observed between any issuance of infant formula by WIC and child obesity risk were expected, given prior publications on the association of lower amounts of formula issued with decreased obesity risk [21, 22, 30]. Two recent meta-analyses found that breastfeeding was robustly associated with lower risk of subsequent child obesity and overweight, and that this association was only partially attenuated by maternal and household factors which are hypothesized to confound the protective association of breastfeeding with child obesity [3, 40]. The association observed between any issuance of a CSSF by WIC and elevated obesity risk was also expected [41], and extend previously published results of the association of CSSF issuance with elevated obesity risk from fully formula fed infants [5] to infants with fewer months of total formula issuance. This also aligns with a higher rate of weight gain observed among infants in a short duration randomized trial of CSSF infant formula [42]. To our knowledge, this is the first study to examine associations of SOYF and ARSF formula types with obesity risk. No association between issuance of SOYF or ARSF and obesity risk was identified relative to the other infant formulas. This was unexpected for SOYF because the primary sugar in the soy-based formula is glucose; however, given the potential for confounding by indication (i.e. families with a history of cow’s milk allergy choosing a soy-based formula) this result should be interpreted cautiously. Further, soy-based infant formula has previously been associated with altered epigenetic modification among females [43], and while no difference in association was observed between male and female children in this study (data not shown) it is possible that soy-based formula may have sex-specific effects that emerge after a longer induction period, or the influences of soy on epigenetic modification of genetic expression offset the influence of a glucose-based formula on growth. The absence of an association between ARSF and elevated obesity risk relative to other formulas was interesting, but not unexpected, given the association between ARSF receipt and reduced spit-up [44] and the potential for confounding by indication (recipients potentially being more likely to have clinically relevant excess spit-up frequency which is associated with reduced growth) [45].

The association between receipt of a CSSF and child obesity relative to other infant formulas was significantly modified by the neighborhood food environment in this population, with greater magnitude of association between CSSF receipt and relative risk of obesity observed in neighborhoods with higher density of healthy food outlets and lower density of unhealthy food outlets (i.e. healthier food environments) than neighborhoods with lower density of healthy food outlets and higher density of unhealthy food outlets (i.e. less healthy food environments). The density of healthy food outlets was previously found to be associated with heavier weight status among WIC-participating children age 2–4 years, with higher weight-for-height z-scores in neighborhoods with high and low density of health food outlets [36]. Another prior study among WIC participant in Southern California identified significantly larger reductions in obesity following policy changes in neighborhoods with healthier food environments [37]. Stronger associations previously reported between health behavior interventions and positive health outcomes among children in less adverse neighborhood environments (lower deprivation, lower crime, healthier food environments) [37, 46, 47] suggests that stronger associations between risk factors (e.g. CSSF receipt) and outcomes (e.g. obesity) may be anticipated in the absence of adverse environmental influences, as was found in the present study. This may be attributable to the less healthy neighborhood environment contributing to obesity risk, independently of infant feeding practices, therefore reducing the magnitude of the association between CSSF receipt and elevated child obesity risk in these less healthy neighborhood environments.

The pronounced racial/ethnic disparities in obesity prevalence observed in this study, with markedly higher obesity observed among Hispanic children relative to non-Hispanic groups and particularly for Asian (57% lower) and White (34% lower) children relative to English-speaking Hispanic children, aligns with racial/ethnic patterns in obesity prevalence observed nationally [12, 48, 49]. These disparities are substantial, were apparent by 24 months of age and for all groups, except Spanish-speaking Hispanic which had dissipated by 48 months of age, persisted at similar magnitude through 60 months of age (data not shown). In national data from the Early Childhood Longitudinal Study Birth Cohort, risk factors for childhood obesity varied between racial/ethnic groups with the highest number of risk factors observed for non-Hispanic Black children and the lowest number observed for Asian children, with risk factors for childhood obesity having different magnitudes of effect in different racial/ethnic groups [12]. In the present study, adjusting for infant feeding practices (i.e. amount and type of formula) eliminated the obesity disparity between English-speaking and Spanish-speaking Hispanic groups, but did not meaningfully change any other observed disparities. Adjustment for neighborhood food environment and Child Opportunity Index did not alter any observed associations, though these results should be interpreted cautiously as the contextual measures used in this study are unlikely to fully capture the differences in neighborhood context and access to resources which may be relevant to group differences in obesity risk. The robustness of the observed racial/ethnic disparities in obesity in this sample to adjustment for neighborhood environment and infant feeding was similar to the Early Childhood Longitudinal Study Birth Cohort results, in which infant feeding (ever breastfeeding and early introduction of solid foods) and neighborhood safety did not significantly contribute to racial/ethnic disparities in obesity prevalence among pre-school age children [12]. It is important to note that children are not born with obesity, and factors which contribute to or reduce the risk of obesity including health behaviors (diet, physical activity, sleep) and stress are a product of individuals with personal characteristics interacting with environments [50–52]. Structural factors, such as redlining, may contribute to differential exposure to obesity-promoting neighborhood environments between groups, constrain opportunities for physical activity [53] and healthy diets [54], and contribute to observed group differences in childhood obesity [53, 54].

CSSF receipt may contribute to obesity risk via metabolic programming by the higher glycemic index of glucose relative to lactose [24, 55], alteration of the infant gut microbiome [25] potentially leading to more rapid infant weight gain [56], or by subsequent differences in diet due to developing dietary preferences [57, 58]. The pattern of effect modification by the neighborhood food environment (i.e. larger relative risk of obesity associated with any CSSF receipt in neighborhoods with healthier food environments) contradicts the expectation for dietary preferences being on the causal pathway, as unhealthy food environments are thought to contribute to higher obesity risk via the relative abundance of unhealthy foods leading to higher consumption of unhealthy foods. This may indicate that the increased obesity risk associated with CSSF receipt is alternatively driven by metabolic programming or alteration of the gut microbiome.

This study has a number of noteworthy strengths, including the large sample with prospectively collected infant feeding information and child anthropometric measurements. Both infant feeding information, based upon WIC infant package issuance data, and child obesity, based upon heights and weights measured by WIC staff, have been previously validated [27, 31]. The study was restricted to children with infant package information across the infant year (0–12 months of age), allowing for characterization of infant feeding and infant formula exposure with prospectively collected data for the first 12 months of life. All analyses of the association between infant formula type and obesity controlled for the amount of formula issued by WIC during the infant year, and were further adjusted for child and household characteristics thought to be potential confounders of the association between infant feeding and obesity. Study limitations include the observational design, precluding causal inference, and the potential for unmeasured confounding including by maternal factors which may contribute to infant feeding decisions, the introduction of complementary foods and beverages, child diet, day care attendance, and physical activity. The study was limited by its use of a static measurement of the density of the food environment in the census tract of residence in 2017, allowing for possible measurement error due to the uncertain geographic context problem where the environmental context that influences dietary behaviors, and subsequently obesity, may not be captured in the census tract of residence or at the time of the food environment assessment [59]; however, prior studies have identified associations between food environment exposures based upon the census tract of residence and obesity outcomes in Southern California WIC participant populations [36, 37]. Despite study restrictions, the sample remained representative of WIC participants in Los Angeles County with regards to race/ethnicity-language preference and household socioeconomic status indicators [60], infant feeding practices [61], and child obesity [62]. The WIC population in this study is predominantly Hispanic, and has high obesity incidence. Caution should be exercised in generalizing the results of this study to populations that are lower proportion Hispanic, higher income, and with lower obesity risk.

## Conclusions

In conclusion, receiving any type of infant formula was associated with higher obesity risk, and any receipt of a CSSF was associated with 5% higher risk of obesity compared to receiving only other types of infant formula among WIC-participating children ages 2–4 years in Southern California. Receipt of SOYF and ARSF formulas was not associated with elevated risk of obesity. The association between CSSF and elevated obesity risk was independent of child factors (including the amount of formula received), household, and neighborhood characteristics that may also contribute to elevated obesity risk. The association between CSSF receipt and elevated obesity risk was stronger among children living in neighborhoods with more healthy food environments, suggesting that the influence of CSSF on obesity risk may function along a pathway independent of subsequent child diet. Racial/ethnic disparities in obesity risk at 2–4 years of age were robust to adjustment for both infant feeding practices (amount and type of infant formula) and neighborhood environment (food environment and Child Opportunity Index), and much of the difference in obesity risk between racial/ethnic groups remained unexplained by infant feeding and contextual factors considered in the present study. Further research is needed to establish whether the observed associations between CSSF receipt and obesity risk are causal, and whether they are mediated by glycemic-programming of the infant metabolism or alteration of the infant gut microbiome. Further research is also needed to identify early life factors that contribute to obesity risk disparities in the WIC participant population in Southern California.

## Data Availability

The data described in the manuscript will not be made available because the data are confidential administrative data of the WIC program. The code book and analytic code will be made available upon request. All requests should be addressed to the Data Mining Project at datamining@phfewic.org.

## Abbreviations

ARSF: Cow’s milk-based infant formula with added rice starch
BF: Breastfed
BMIz: Body mass index z-score
CI: Confidence interval
CFB: Complementary foods and beverages
CSSF: Lactose-reduced infant formula with corn syrup solids
EN: English-speaking
INF: Infant feeding
LAC: Los Angeles County
NCon: Neighborhood context
RR: Risk ratio
SOYF: Soy-based infant formula
SP: Spanish-speaking
WIC: The Special Supplemental Nutrition Program for Women, Infants, and Children
